# Assessment of Pediatric Residents’ Attitudes toward Anticipatory Counseling on Gun Safety

**DOI:** 10.3390/children6110122

**Published:** 2019-11-01

**Authors:** Daon D. Juang, Diane L. McDonald, Elizabeth A. Johnson-Young, Tierra D. Burrell, Dana L. Silver, Yan Wang, Richard Lichenstein

**Affiliations:** 1 Department of Pediatrics, University of Maryland School of Medicine, 655 West Baltimore Street, Baltimore, MD 21201, USA; dianemcdonaldmd@gmail.com (D.L.M.); yan.wang@som.umaryland.edu (Y.W.); RLichenstein@som.umaryland.edu (R.L.); 2 Department of English, Linguistics, and Communication, University of Mary Washington, 1301 College Avenue, Fredericksburg, VA 22401, USA; ejohnso6@umw.edu; 3 Department of Pediatrics, Kaiser Permanente Mid-Atlantic Permanente Medical Group, 2101 East Jefferson Street, E East, Rockville, MD 20853, USA; Tierra.D.Burrell@kp.org; 4 Department of Pediatrics, The Herman and Walter Samuelson Children’s Hospital at Sinai, 5051 Greenspring Avenue, Baltimore, MD 21209, USA; Dsilver@lifebridgehealth.org

**Keywords:** Asking Saves Kids (ASK), firearm safety, firearm deaths, gun ownership, anticipatory guidance

## Abstract

Introduction: Guns remain a major cause of injury and death among children. We determined pediatric residents’ familiarity with gun safety campaigns and their gun safety counseling practices. We determined pediatric residents’ comfort with the Asking Saves Kids (ASK) campaign, which recommends that parents ask about gun safety and storage where their children play. Methods: Cross-sectional 27-item electronic survey was distributed to three pediatric residency programs in Baltimore, Maryland, USA. Residents were asked to respond to statements using a seven-point Likert scale on familiarity with three gun safety campaigns and their attitudes toward gun safety counseling. Results: 82% of respondents were not familiar with gun safety programs. 23% reported not counseling. 87% believed it is a good idea to ask about guns in a home but only 64% were comfortable recommending their patients’ parents to ask about guns. 59% were personally comfortable asking about guns in the home. 15% believed their patients’ parents would be comfortable asking about guns in the homes of friends and families. Conclusions: The residents in this survey supported the idea of gun safety anticipatory guidance but discussing firearms can be problematic. Educational programs and strategies are needed to support physicians’ counselling on gun safety.

## 1. Introduction

The Centers for Disease Control and Prevention reported that firearms are the second leading cause of death for children over the age of 1 in the United States [1]. At routine well-child visits, pediatricians address a variety of safety topics to reduce injury and death through the Bright Futures program, a health and prevention initiative, which also includes gun safety [2]. The American Academy of Pediatrics (AAP) recommends that pediatricians discuss safe gun storage with parents as part of their anticipatory guidance [3]. As stated in the AAP policy statement on firearm-related injuries, safe gun storage (i.e., having guns unloaded and locked and storing ammunition separately in locked locations) reduces accidental injury and suicide risk in children and adolescents [3].

In order to address the safety concerns, there are multiple initiatives aimed to reduce firearm injury and death. The AAP collaborated with the Brady Center to Prevent Gun Violence and developed the Asking Saves Kids (ASK) campaign, which encourages parents to ask relatives and friends whether there is an unlocked/unsecured gun in their home before sending their child over to play [4]. Be SMART, supported by Everytown for Gun Safety and Moms Demand Action for Gun Sense in America, like ASK, recommends that parents question the gun safety and storage practices of relatives and friends in whose homes their children may be playing but also acknowledges the role of firearms in suicide and encourages additional protective measures [5]. The Eddie Eagle GunSafe Program, sponsored by the National Rifle Association, is different from ASK and Be SMART in that it speaks to children, not their parents, advising children to not touch a weapon if they see one and to run away from the location and tell a grownup [6].

Despite recommendations for physicians to counsel their patients about the risks, only half of the pediatric medicine trainees participating in a survey conducted by Solomon and colleagues, reported counseling routinely and 20% never counseled [7]. It is important to understand how pediatric trainees now address gun safety in their practices.

Given the need for gun safety counseling by pediatricians, this study, therefore, aimed to determine pediatric residents’ familiarity with gun safety campaigns in Baltimore, Maryland, USA. In order to understand potential barriers toward counseling, we assessed residents’ attitudes and comfort level regarding the provision of anticipatory guidance, including asking about firearms and firearm storage in another home before sending children there to play. Understanding attitudes and comfort toward counseling during residency is important in how pediatricians will ultimately independently practice.

## 2. Materials and Methods

We conducted a cross-sectional electronic survey of all pediatric residents via email in the Baltimore, Maryland, region using Survey Monkey^®^. They were enrolled in residencies at University of Maryland Children’s Hospital, Johns Hopkins Children’s Hospital and The Herman and Walter Samuel Children’s Hospital at Sinai. The residency programs had tracks for categorical pediatrics, internal medicine–pediatrics, emergency medicine–pediatrics, pediatrics–genetics and pediatrics–anesthesia, and were active during the 2017–2018 academic year. The survey opened in August 2017 and closed in October 2017. Participation was voluntary and the names of those who completed the survey were placed into a draw for Amazon gift cards. The survey was approved by the institutional review boards at the University of Maryland School of Medicine, Johns Hopkins School of Medicine and Sinai Hospital.

### 2.1. Survey Data

A 27-item online survey was developed by the authors to assess residents’ knowledge, clinical practice, attitudes and comfort regarding firearms and gun safety programs. The survey was developed de novo based on expert consensus based on themes. Authors confirmed questions and responses but there was no resident testing of the items prior to implementation. We collected the following demographic data: program, year of training, gender and gun ownership status.

We asked participants about their familiarity with and attitudes toward national gun campaigns that advocate inquiry into patients’ access to firearms and parents’ willingness to ask about firearm storage in another home. Respondents’ awareness of the ASK campaign, the Be SMART campaign and the Eddie Eagle GunSafe Program was determined by asking them to choose the relevant comment: (1) Yes, I recommend this; (2) No, I do not recommend this; or (3) I am not familiar with this program.

Residents were asked about their clinical practice regarding counseling on gun safety during well child visits. They were asked if they offer such guidance and, if so, how they do it. Do they use pamphlets/handouts and engage in discussion? Do they rely on pamphlets or handouts only? Do they use discussion only? Or do they not counsel parents on firearm safety?

The residents’ attitudes toward asking about firearms and firearm storage in another home were determined using a seven-point Likert scale that offered the following range of answers: strongly agree, agree, somewhat agree, neither agree or disagree, somewhat disagree, disagree and strongly disagree. They were asked to respond to the following statements: (1) Anticipatory guidance should include firearm safety; (2) It is a good idea to recommend that parents ask about guns and gun storage in another home; (3) Pediatricians should identify patients with access to guns outside their homes (e.g., daycare, playmates, relatives); and (4) A parent’s view on gun control impacts my guidance on firearm safety.

The residents were also asked to describe their level of comfort toward asking about firearms and firearm storage in another home. Using the same seven-point Likert scale, they were asked to respond to the following statements: (1) I am comfortable recommending that parents ask about guns and gun storage in another home; (2) I am personally comfortable asking about guns and gun storage in another home; and (3) My patients’ parents are comfortable asking about guns and gun storage in another home.

### 2.2. Statistical Analysis

Descriptive analyses were conducted for demographic variables. Frequencies for the responses were estimated.

## 3. Results

We distributed surveys to all 181 pediatric residents in the region (71% in categorical programs and 29% in combined programs). The response rate was 60% (*n* = 108). Respondents’ demographics are categorized in Table 1. There was no significant difference in gender or years of training between survey responders and non-responders (data not shown). Two of every three respondents were female, they all were younger than 45 years of age and very few of them (6/108) owned a firearm.

The residents’ familiarity with national firearm safety campaigns is summarized in Table 2. Most of them were not familiar with the three most popular programs (82–94%). Recommendation rates were low; the ASK program was recommended most often.

The residents’ clinical practice in regard to offering anticipatory gun safety guidance is summarized in Table 3. Almost one-fourth of the respondents did not counsel parents about firearm safety. For those who did talk with parents about gun safety, the primary form of communication was discussion.

The residents’ attitudes toward asking about guns and gun storage practices in someone’s home before sending a child over to play are displayed in Table 4. Ninety-six percent of them believed that anticipatory guidance should include firearm safety. Specifically, 87% of the respondents believed it is a good idea to ask about guns and gun storage. About half reported that the parents’ view of gun control influenced their decision to provide anticipatory guidance.

Residents’ degree of comfort in asking about guns and gun storage practices in someone’s house before sending a child to play is portrayed in Table 5. Sixty-four percent of them reported being comfortable with recommending that parents ask about guns and gun storage in another home before sending their children over to play. Fifty-nine percent were personally comfortable about asking about guns and gun storage. Only 15% of trainees believed their patients’ parents are comfortable with asking about guns and gun storage in another home.

## 4. Discussion

Despite the continuing problem of gun violence and the positive impact of counseling in the office setting, we found low levels of familiarity with gun safety anticipatory campaigns for pediatric trainees, and problems in the discussion of the guns and gun storage in another home.

Our study group was young and predominantly female and did not own firearms. This profile matches that of the pediatrics workforce [8]. There were no differences in firearm counseling practices and attitudes based on residency training program, age, or sex of the trainee. The fact that very few residents surveyed owned a gun is similar to the findings of Olson and colleagues [9].

Despite the promotion of the ASK campaign for more than 10 years, the majority of residents were not familiar with this AAP program. Most of the residents in our survey were also not aware of three national firearm safety programs.

The primary method of firearm safety anticipatory guidance was by discussion in the office. Handouts and pamphlets were not commonly used but may be useful for parents to have as a later reference. In other forms of anticipatory guidance, written information alone has not been effective for prevention of other injuries [10]. We did not ask respondents about the content of the discussions or about the exact materials they distributed to verify their accuracy or content. In addition, among those who reported counseling on firearm safety, the frequency and timing of firearm safety discussion was not asked.

Almost 1 out of 4 of the residents in our study group said they do not counsel on gun safety. Despite the AAP’s promotion of the ASK campaign, this is similar to survey findings in 2002 where 20% of pediatricians did not counsel [7]. Our study attempted to investigate attitudes and comfort toward gun safety anticipatory guidance, but other barriers may exist such as finding time in the visit to address gun safety with the large amount of other expected anticipatory guidance [11].

The vast majority of our study group acknowledged the importance of anticipatory guidance regarding firearm safety. In contrast, a similar study conducted in Maryland in 1992 found that 74% of pediatricians thought they had a responsibility to counsel families on firearms [12]. The increase over the intervening 20 years might reflect the increasing number of firearm-induced catastrophes and the awareness that firearm injury is preventable and a priority.

Despite almost all residents acknowledging the importance of firearm anticipatory guidance, a little over half of the respondents are comfortable with making the recommendation that parents ask about guns and gun storage before sending their child over to play. One of the potential barriers is a parent’s view on gun control. Slightly more than half of residents said a parent’s views on gun control affected their decision to offer anticipatory guidance. A physician’s desire to provide information about gun safety might be thwarted by concerns about alienating a parent who is a gun owner. Garbutt and colleagues found that parents who own guns are less likely to be receptive to such screening than parents who do not own guns (58% vs. 71%) [13]. State legislation may also be a factor in a pediatrician’s ability to counsel on gun safety related issues. As recently as 2011, Florida State Legislature passed the Privacy of Firearms Act, which threatened large fines or even loss of medical licensure if pediatricians asked families about gun ownership and habits [14]. Although it was overruled by the 11th United States Circuit Court of Appeals, pediatricians may feel the threat of repercussions regarding gun safety discussions with the parents of their patients [15].

The minority of respondents felt that their patients’ parents would be comfortable asking other parents about their guns and gun safety practices. We did not interview parents, so it is unknown if patients’ parents truly would be comfortable about asking about guns in the homes of friends and family. Since only more than half of pediatricians themselves are comfortable asking this question, one would suspect that this discussion may be problematic for all. Strategies for a gun safety dialogue that acknowledges potential barriers of talking about gun safety, but then includes the provider’s personal concerns about their own children’s safety, their child’s ability to get into mischief, and news reports of unintentional shootings in homes, may be helpful in promoting discussion regarding firearm and ammunition storage [16].

A multifactorial toolkit for gun safety anticipatory guidance may be ideal for resident training. Pediatric residents need to be educated about how guns work, gun safety campaigns, and effective interventions to decrease unintentional and intentional gun injuries. Tools like the ASK campaign are a start but based on our findings, need to be supplemented with instruction on motivational interviewing on how to counsel families to routinely ask their patient’s parents about the presence and security of firearms in another’s home. The AAP needs to support the ASK program so that awareness is at least 90% and residents feel comfortable on how to use it. This can be supported with motivational interviewing instruction as detailed above, sample discussions and handouts.

A major limitation of the study included the survey instrument not being validated. We did not see validated tools in the literature or use consensus methodology to explore important themes regarding gun safety counseling. Survey responses were self-reported, so bias might have been introduced, reflecting resident’s expectations instead of their actual practice. We also did not look or compare the curriculum that the pediatric residency programs used, which may have influenced responses but there were no statistically significant differences between individual residency programs. In addition, respondents represented pediatric trainees from one metropolitan region and may not represent the views of those around the rest of the country.

## 5. Conclusions

Despite the increased prevalence of pediatric firearm deaths and injuries, there has been little progress in firearm safety counseling. Most pediatric residents we surveyed were not familiar with national gun safety programs. Although most of them believe that anticipatory guidance should include firearm safety, almost one-fourth of them do not discuss gun safety measures with their patients’ parents. The vast majority of respondents believe it is important to ask about the presence and storage of guns in someone else’s home, but only half of them would feel comfortable asking a neighbor or relative themselves; almost no resident felt that their patient’s parents would be comfortable broaching this question. Strategies are needed to promote the topic of gun safety and to facilitate residents’ ability to discuss it during patient visits.

## Figures and Tables

**Table 1:** Demographics of respondents.

	No. (%)
Gender	
Male	34 (31)
Female	74 (69)
Gun ownership	
Yes	6 (6)
No	102 (94)
Year(s) from medical school graduation	
0	31 (29)
1	29 (27)
2	27 (25)
3+	19 (18)
Age	
<45	108 (100)
≥45	0 (0%)

**Table 2:** Residents’ familiarity with gun safety campaigns.

Campaign	Recommend	Do Not Recommend	Not Familiar
Eddie Eagle	3%	4%	94%
Be SMART	5%	3%	92%
ASK	15%	3%	82%

**Table 3:** Residents’ clinical practice regarding anticipatory guidance for firearm safety.

Method	Percentage
Pamphlets/handouts and discussion	6
Pamphlets or handouts only	0
Discussion only	71
I do not counsel on firearm safety.	23

**Table 4:** Residents’ attitudes toward firearm safety counseling.

Statement	Percentage That Agreed
Anticipatory guidance should include firearm safety.	96
It is a good idea to recommend that parents ask about guns and gun storage in another home.	87
A parent’s view on gun control impacts my guidance on firearm safety.	55

**Table 5:** Residents’ comfort in regard to asking about guns and gun storage practices in someone’s house before sending a child over to play.

Statement	Percentage That Agreed
I am comfortable recommending that parents ask about guns and gun storage in another home.	64
I am personally comfortable asking about guns and gun storage in another home.	59
My patients’ parents are comfortable asking about guns and gun storage in another home.	15
